# Calcium and Non-Penetrating Traumatic Brain Injury: A Proposal for the Implementation of an Early Therapeutic Treatment for Initial Head Insults

**DOI:** 10.3390/biom14070853

**Published:** 2024-07-15

**Authors:** Danton H. O’Day

**Affiliations:** 1 Department of Biology, University of Toronto Mississauga, Mississauga, ON L5L 1C6, Canada; danton.oday@utoronto.ca; 2 Cell and Systems Biology, University of Toronto, Toronto, ON M5S 3G5, Canada

**Keywords:** traumatic brain injury, concussion, calcium dysregulation, calcium ion channels, calcium-binding proteins, calmodulin, calcium-dependent inactivation, sports-related TBI, therapeutic proposal, post-concussive syndrome

## Abstract

Finding an effective treatment for traumatic brain injury is challenging for multiple reasons. There are innumerable different causes and resulting levels of damage for both penetrating and non-penetrating traumatic brain injury each of which shows diverse pathophysiological progressions. More concerning is that disease progression can take decades before neurological symptoms become obvious. Currently, the primary treatment for non-penetrating mild traumatic brain injury, also called concussion, is bed rest despite the fact the majority of emergency room visits for traumatic brain injury are due to this mild form. Furthermore, one-third of mild traumatic brain injury cases progress to long-term serious symptoms. This argues for the earliest therapeutic intervention for all mild traumatic brain injury cases which is the focus of this review. Calcium levels are greatly increased in damaged brain regions as a result of the initial impact due to tissue damage as well as disrupted ion channels. The dysregulated calcium level feedback is a diversity of ways to further augment calcium neurotoxicity. This suggests that targeting calcium levels and function would be a strong therapeutic approach. An effective calcium-based traumatic brain injury therapy could best be developed through therapeutic programs organized in professional team sports where mild traumatic brain injury events are common, large numbers of subjects are involved and professional personnel are available to oversee treatment and documentation. This review concludes with a proposal with that focus.

## 1. TBI: Its Initiation and Long-Term Effects

Worldwide, an estimated 64 to 74 million cases of traumatic brain injury (TBI) occur annually and there is evidence the incidence of TBI is increasing [1,2,3]. The diverse causes, biomarkers, pathophysiology and personal and social impact of TBI have been well studied and reviewed [3,4,5,6,7,8]. Despite TBI being a major cause of disability and death internationally, therapeutic attempts so far have failed to generate significant help [7,9]. Part of this may be due to its complicated physiopathology that results from the innumerable factors that instigate TBI and the complexity of secondary physiological insults. For example, TBI can be initiated by a simple fall, a hit to the head by another person (e.g., during team sports) or an object (e.g., baseball, hammer, bullet) or by multiple other causes. These reflect two basic classifications of TBI: closed head injury (CHI), with no skull fracture, and penetrating brain injury (PBI) which involves skull fracture [10]. Of course, all cases of TBI typically begin with a primary initiating event.

**Figure 1:**
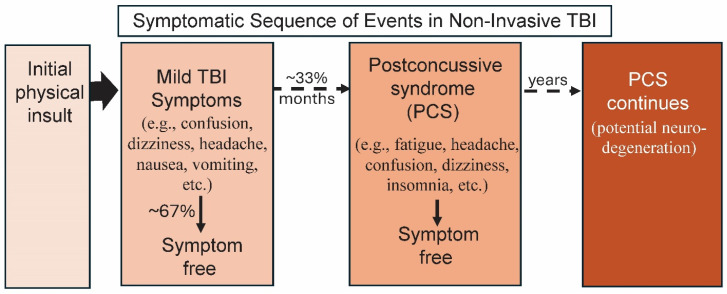
Early and subsequent symptomatic events linked to non-invasive, mild TBI. In mild non-invasive TBI, the initial physical insult leads to the initial mild TBI symptoms. While symptoms can vary widely, they can include loss of consciousness, dizziness, headache, nausea and vomiting. Over the next few months, around two-thirds of cases will be symptom-free while the remaining patients will progress to the more serious post-concussive syndrome (PCS). PCS individuals will continue to suffer with a wide diversity of symptoms that may include fatigue, continued headaches, confusion, dizziness along with insomnia and/or other symptoms. With time, most PCS patients become symptom-free. After years or decades, some PCS cases progress to neurodegenerative diseases such as Alzheimer’s disease.

Mild TBI (mTBI), a type of CHI, is the simplest form of TBI yet it accounts for 80–90% of all emergency department TBIs [11]. mTBI is also called concussion. Resulting from a bump, knock, jolt or other type of blow to the head, it is non-penetrating but can result in a loss of consciousness or altered consciousness usually lasting less than 30 min (Figure 1) [12,13]. mTBI is also associated with short-term dizziness, headache, impaired memory, nausea and vomiting, among other symptoms. Most cases are transitory, and the initial symptoms disappear but on rare occasions brain bleeding or swelling has resulted in death within 24–48 h. The chance of disability or death is greatly increased if a second concussive incident happens within a few weeks after the first [14]. Approximately one-third of mTBI cases will suffer from secondary symptoms over several months: fatigue, difficulty concentrating, headache, confusion, dizziness, insomnia and vision change, among others [15]. These and other common post-concussive syndrome (PCS) symptoms often resolve within another week or two but can persist for longer. Decades later TBI can lead to neurodegenerative diseases including Alzheimer’s disease (AD), Parkinson’s disease (PD) and other forms of dementia [16].

While TBI is a major cause of death and disability internationally, there is a lack of effective therapeutic treatments [7]. Several inter-related issues have previously confounded the development of effective treatments for TBI including but not limited to disease heterogeneity, the lack of standardized protocols in model studies plus inter-collaboration and data replication [17]. About 6% of emergency room (ER) visits are a result of sports-related mTBI [18]. Sports-related mTBI ER visits correlate well with progression to PCS, but this area has not been well studied. A study of 4360 individuals revealed that persistent impairment is a significant outcome of sports-related TBI, even when the initial insult was considered mild. Based on their results from imaging and immunostaining for neurodegenerative biomarkers in the brains of rats subjected to either one or three successive concussive brain impacts, McDaid and colleagues concluded “even a single concussive event is sufficient to elicit long-term synaptic effects” and “detrimental long-term cognitive symptoms” [19]. These results argue for initiating therapeutic measures as early as possible after an initial brain impact and organized sports may offer the best route to developing such therapies since current approaches are not effective.

## 2. Current Treatments for mTBI

A special issue of *Neurotherapeutics* published in 2023 covers advancements in the treatment of TBI. Summarizing the contents of each article, the editors indicate there are arguments that the past few decades of research have not been successful in finding a successful treatment for TBI but a new third wave of research will build on the past with new approaches including neuroimaging and biomarker analysis [20]. There is no doubt such work is essential for developing therapies for progressive TBI but overall, the multi-article issue fails to address the initial stages of TBI that literally occur within seconds or minutes of the primary trauma. According to Syzdykbayev and colleagues, “Over the past 20 years, more than 30 traumatic brain injury (TBI) guidelines have been developed and updated by various organizations [21]. However, there is currently no ultimate and effective guideline or treatment protocol for TBI”. When conditions are moderate to severe, then an initial assessment using a non-contrast computed tomography (CT) scan becomes the standard of care [22]. While considered as a “quick” assessment, this requires transport to a CT facility, access to technicians and equipment that may or may not be available followed by the scan itself after which one or more days are needed to analyze the results. In total, the average person’s condition might not be assessed for many days during which time their condition could continue to deteriorate.

The initial treatment of mTBI cases is essentially nonexistent despite compelling evidence that many cases progress to serious symptoms. According to the Mayo Clinic website, “Mild traumatic brain injuries usually require no treatment other than rest and over-the-counter pain relievers to treat a headache” [23]. This opinion is expressed on other legitimate medical websites including the National Institute of Child Health and Human Development (NICHD), “Mild TBI, sometimes called concussion, may not require specific treatment other than rest”. These are just two examples of many of the top medical resources that suggest, unless there are serious obvious initial symptoms, the only treatment for mTBI is rest. While the goal is to let the brain heal, when not coupled with any therapeutic intervention, this only allows time for the negative events caused by the initial insult to continue causing damage. Considering around one-third of mTBI cases can progress to the serious symptoms associated with PCS, this lack of intervention immediately after the initial mTBI insult is poor medicine. Since it is impossible to predict the ultimate result from an initial mTBI event and since those results could not only be life-altering but lethal, then it seems logical that therapeutic intervention should begin as early as possible. The earlier therapeutic treatment begins, the greater the chance mTBI will not lead to PCS. One initial event resulting from mTBI is the disruption of intracellular calcium signalling, thus presenting multiple targets for therapeutic intervention.

## 3. Calcium Dysregulation and TBI

The initial mTBI head insult results in tissue deformation and necrotic death of neurons, microglia and other brain cells leading to altered blood flow, osmotic and electrolyte imbalance including dysregulation of calcium levels [4,5,24]. It also begins with the disruption of the blood–brain barrier (BBB), cell stretching, shearing and necrosis, events that initiate a secondary cascade of neuroinflammation, mitochondrial dysfunction and cell death. Over time, changes in cognition and behavior (e.g., anxiety, aggression, depression, loss of memory, personality changes) can develop with links to neurodegenerative diseases including Parkinson’s and Alzheimer’s [16,25]. Of interest here is the early and initial and critical disruption of calcium levels of TBI since it presages many of the progressive, longer-term, harmful pathophysiological effects including neuroinflammation and mitochondrial dysfunction that can progress to neurodegeneration.

**Figure 2:**
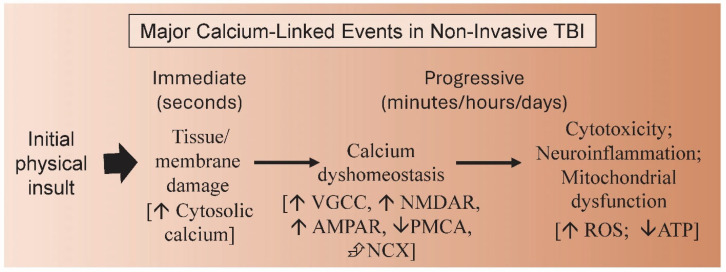
The immediate and progressive calcium-linked events of mTBI. An initial physical insult to the head causes tissue and cell damage causing, among other events, membrane shearing and tears that allow the unregulated influx of calcium ions from the cerebrospinal fluid (CSF) into cells. This initial increase in intracellular calcium coupled with the effects of membrane shearing and stretching, also disrupts cytosolic calcium homeostasis especially through the increased influx of calcium ions via ion channels (NMDAR, AMPAR). Coupled with this is a decrease in PMCA and reversal of NCX channel activity slowing down the normal release of intracellular calcium to the CSF. Continued calcium dyshomeostasis can lead to cytotoxicity, neuroinflammation and mitochondrial dysfunction, the latter leading to an increase in ROS and drop in ATP production. If unchecked these events can lead to neurodegeneration. Acronyms and symbols: ↓, decrease; ↑, increase; ⮵, reversal; AMPAR, α-amino-3-hydroxy-5-methyl-4-isoxazolepropionic acid receptor; CSF, cerebrospinal fluid; NCX, Na^+^/Ca^2+^ exchanger; NMDAR, N-methyl-D-aspartate receptor; PMCA, plasma-membrane Ca^2+^-ATPase; VGCC, voltage-gated calcium channel.

In humans, the events of TBI have been revealed through the biochemical analysis of bodily fluids and brain imaging techniques (e.g., computed tomography (CT) scans; magnetic resonance imaging (MRI)) hours, days or weeks after the initial traumatic insult [10]. However, insight into the earliest events of TBI has only been revealed through animal research. Understanding the earliest events of calcium dysregulation has only been possible through such studies. For example, after a mild concussive event, intracellular calcium levels were measured in CA3 hippocampal neurons from Sprague–Dawley rats revealing that they were significantly elevated within one day but typically returned to normal levels within a month [26]. However, in some cases disruptions to normal calcium homeostasis were still observed one month after TBI, contributing to long-term symptomology. The first increases in calcium were linked to the damage caused by the initial TBI insult (i.e., tissue damage, membrane leakage) while progressive changes in the divalent cation were due to further disruption of calcium homeostasis mechanisms (i.e., ion channels and receptors) that, in turn, are involved in subsequent complex events (i.e., neuroinflammation, mitochondrial dysfunction) that if maintained could lead to neurodegeneration (Figure 2). The inflammatory response due to stroke has been well studied in rats [27,28]. While the exact timing and sequence of these events in human TBI need to be resolved, Figure 2 presents a useful sequence for understanding the role of calcium in its onset and progression.

The long-term disruption of calcium levels contributes to the morbidity and mortality of TBI and this disruption begins early after an initial mTBI event. As a result of membrane damage permitting calcium leakage into neurons coupled with entry via cell membrane and intracellular ion channels, increased levels of cytosolic calcium occur within seconds of the initial trauma [7,29]. The initial brain insult results in shearing and stretching of neurons coupled with the opening of unregulated pores in brain membranes that allow the uncontrolled influx of calcium and other ions. After that, within 30 min or so, the increased intracellular calcium induces glutamate and other neurotransmitter release stimulating NMDA and AMPA receptors adding further to increased calcium levels which can lead to neurotoxic levels with neuroinflammation and other long-term harmful effects [26].

## 4. Calcium Ion Channels and TBI

**Figure 3:**
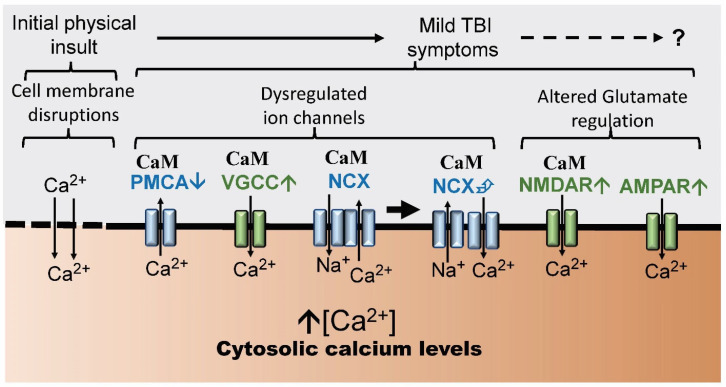
Sources of dysregulated calcium levels in mild traumatic brain injury (TBI) and post-concussive syndrome (PCS). The initial physical insult leading to mTBI can result in cell membrane disruptions that allow the unregulated influx of calcium ions into the neuronal cytosol. The function of regulated ion channels (e.g., VGCC, PMCA, NCX) is also disrupted early on potentially augmenting the increased cytosolic calcium levels. Continued disruption of calcium signaling involving NMDAR and AMPAR along with other events (e.g., neuroinflammation) contribute to the symptoms of PCS. These events are detailed in the main text. Calmodulin (CaM) binding to specific channels is indicated and discussed in the body of the text. The gradient is intended to reflect the increased calcium levels linked to the onset and potential progression of mTBI to PCS but the details of these increases in calcium remain to be elucidated. Acronyms and symbols: ↓, decrease; ↑, increase; ⮵, reversal; ER, endoplasmic reticulum, NCX (Na^+^/Ca^2+^ exchanger), NMDAR (N-methyl-D-aspartate receptor), PMCA (plasma-membrane Ca^2+^-ATPase), VGCC, (voltage-gated calcium channel).

Dozens of ion channels, receptors and calcium-binding proteins are involved in the regulation of calcium homeostasis and dyshomeostasis in the normal brain and during neurodegenerative diseases such as AD, HD and PD [30,31]. To date, only a small number of these regulators have been identified as being critical to the initial and subsequent events of TBI: NCX (Na^+^/Ca^2+^ exchanger), NMDAR (N-methyl-D-aspartate receptor), PMCA (plasma-membrane Ca^2+^-ATPase) and VGCC (voltage-gated calcium channel) (Figure 3). Calcium dysregulation occurs in both the primary/initial traumatic brain injury event as well as in the resulting secondary symptoms. Over 30 years ago, a review revealed a central link between calcium dyshomeostasis in the initial and subsequent events of TBI [24]. In response to a head insult, cerebrospinal fluid (CSF) calcium levels decrease from 1 mM to 0.01 mM as brain tissue calcium levels concomitantly increase [24,32]. This initial unregulated calcium influx results from multiple sources. First, membrane tearing and shearing opens holes in the cell membrane while mechanical strain and stretching activates certain channels including voltage-gated sodium channels which by causing membrane depolarization then activates previously quiescent voltage-gated calcium channels (VGCCs) [33,34]. Normally PMCA and NCX counteract large neuronal cytosolic Ca^2+^ variations by directing Ca^2+^ extrusion from the cytosol to the extracellular milieu [35]. However, mTBI leads to decreased PMCA function and a reversal of NCX ion exchange thus decreasing extracellular calcium release [36,37]. While VGCCs lead to a greater influx of calcium ions, the decreased action of PMCA and the reversal of NCX function decreases their extracellular release, thus augmenting the harmful high calcium ion levels caused by the original damage generated by the primary mTBI insult further driving any calcium-mediated pathophysiological changes.

Membrane depolarization also stimulates the release of the neurotransmitters glutamate and acetylcholine which have been found to occur in response to TBI [38,39]. The release of the excitatory amino acid glutamate leads to an overactivation of glutamate receptors that also add to the levels of cytosolic calcium [40]. Glutamate increases the toxic level of calcium in neurons by binding to and activating mGlu receptors that, through the release of inositol 1,4,5 trisphosphate (IP3) drives the release of calcium ions from the endoplasmic reticulum via IP3 receptors. Continued abnormal calcium levels driven by these additional events have multiple secondary effects including damage to mitochondria, white and grey matter, neuronal degradation and death as well as other problems [41]. High levels of calcium cause mitochondrial damage affecting ATP synthesis and releasing additional toxic levels of calcium [42]. With time, the pathophysiology of TBI becomes increasingly complex not only involving the increasing impact of neuroinflammatory-related events (e.g., autophagy, necrosis) and mitochondrial dysfunction (e.g., decreased ATP, increased ROS) but also neuron degeneration and death as well as continued damage to the blood–brain barrier (BBB) [5,43]. While the initial events of TBI are comparatively limited, with the progression to PCS, the number of cellular processes and events increases exponentially making it difficult to define which of them are most critical leaving the search for therapies more daunting.

The data argue that targeting the calcium dysregulation invoked by an initial head insult is a strong route for therapeutic intervention. However, the road to developing therapies has not been simple. In 2003, a review of the results from six clinical trials indicated there was no evidence for a statistically significant benefit from treating TBI patients with calcium channel blockers [44]. However, ongoing work has shown some promise. Current work suggests N- and L-type channel blockers could be useful in treating TBI with the L-type drug nimodipine offering neuroprotection especially for traumatic subarachnoid hemorrhage but with no significant effect on symptomology or rate of death [21,45,46]. The N-type calcium channel blocker SNX-185 is neuroprotective when administered within one day after TBI directly to the CA2 and CA3 regions of the hippocampus in rats [47]. The potential importance of L- and N-type VGCCs in TBI was again demonstrated with results of treatments with specific channel antagonists in vitro which reduced calcium loads and cell death and with animal models where cell death was reduced, and cognition was improved [48]. Despite the potential importance of L-type and N-type VGCCs, at present there are no established therapeutic guidelines for the use of selective calcium channel blockers for these VGCCs in the treatment of TBI [22]. The lack of evidence supporting the use of calcium channel blockers could be a result of the late timing employed in treatment protocols. The initial cytotoxic influx of calcium ions immediately upon initial head trauma sets off a complex cascade such that later attempts to lower calcium levels could become ineffective because of the over-riding deleterious effects of these changes (e.g., neuroinflammation, glutamatergic effects, mitochondrial dysfunction, etc.).

## 5. The Downstream Role of Calcium Signaling: Calcium-Binding Proteins

Young’s review noted some of the important functions that the activation of downstream calcium signalling mediate and how calmodulin (CaM) could function in response to calcium dysregulation caused by TBI [24,49]. Noting the failure of therapies targeting sources of calcium influx, Young wrote: “Drugs aimed at specific intracellular Ca^2+^ signaling pathways or subcellular components show some promise for the future treatment of TBI, as well as other disease states of the brain such as neurodegenerative disorders”. The impact of calcium dysregulation is largely due to its effects on downstream calcium-binding proteins (CBPs), an area in which there has been comparatively little study related to TBI. Studies on the use of the CBP S100 as a biomarker for TBI have not supported its value as an indicator of head trauma [50]. On the other hand, the work that has been carried out has shown that in response to a TBI insult, increased cytosolic calcium levels drive the activation of calpain and CaM [51,52,53]. Elevated calcium levels induced by TBI activate cysteine proteases including calpains which degrade a diversity of targets causing neuron damage [54]. Calpain, which is activated by calcium ions, increases in activity within minutes of injury with the increased levels being maintained for subsequent days [55]. This family of calcium-activated proteases, especially calpain-2, also functions in multiple neurodegenerative diseases [30]. An article on calpain-1 and -2, in a 2023 special issue of *Neurotherapeutics*, deals with the effects of the increase in cytosolic calcium caused by TBI but the cited research involves downstream events with later implications rather than being related to the initial phases of TBI [56]. The results of previous studies using calpain-1 and -2 inhibitors for the treatment of TBI have been contradictory likely not only because of the different substrates for each enzyme and their specific biological functions but also due to the timing of their therapeutic use. That said, there is evidence that newly developed calpain-2 inhibitors (e.g., NA-184) may be useful in the treatment of “the long-term pathological consequences of seizure activity” [56]. Still, the role of calpains in the initial events of TBI and their potential value as therapeutic targets remains to be resolved.

## 6. Calmodulin-Regulated Ion Channels and TBI

Calcium dysregulation is an early event in mTBI and in addition to calpains, CaM is a primary target and effector for this divalent cation [51,52]. CaM senses cytosolic levels of calcium and regulates them via binding to specific channels and transporters. Neurons in the mammalian CNS have an extremely high level of CaM (10–100 mM) that is involved in binding to and regulating over five dozen kinases, ion channels and downstream signaling events critical to neuronal function and survival [52,53]. Thus, understanding CaM’s involvement could lead to new therapeutic targets. Expressed at high levels in neurons, CaM is a small (148aa; 16.7 kDa) highly conserved protein [57,58]. Essential for the function and survival of all eukaryotic cells, CaM controls numerous, critical neuronal functions including exocytosis, ion channel function, neurotransmission, synaptic strength, learning and memory, as well as plasticity. To do this, it binds CaM-binding proteins (CaMBPs) [59]. It is able to do this via unconventional calcium-dependent binding domains (CaMBDs) in its target CaMBPs, events that have been well studied and reviewed [60,61,62,63,64]. In the absence of calcium ions, apoCaM is a comparatively compact protein that binds to CaMBPs via IQ, IQ-like or unconventional IQ variants [65]. However, in the presence of calcium apoCaM immediately undergoes a remarkable conformational change resulting in an extended Ca^2+^/CaM configuration and exposing critical hydrophobic target-binding amino acids. It is likely that this transformation of apoCaM to Ca^2+^/CaM is an initial event in TBI since the increase in calcium caused by tissue damage would immediately enforce this transformation.

The role of CaM in the regulation of critical calcium ion channels in neurodegenerative diseases has been well reviewed [30,31]. There are extensive data revealing a potential and central role for CaM in the events of TBI, since CaM binds to and differentially regulates the following critical channels: NCX, NMDAR, PMCA and VGCC (Figure 3). PMCA is involved in the early events of TBI [36,37]. It is a P-type Ca^2+^-ATPase superfamily member that directs the efflux of calcium ions from the cytosol to the extracellular environment [66]. CaM is a primary cellular regulator of PMCA. Neuronal PMCAs possess a regulatory C-terminal autoinhibitory domain that prevents calcium efflux by keeping the pump inactive at low calcium levels [67]. With an increase in cytosolic calcium, Ca^2+^/CaM binding to this regulatory sequence in PMCA relieves this inhibition causing an increase in calcium efflux [68].

The activation of NMDARs as a result of TBI-induced excitotoxicity is an appealing but challenging target for overcoming some of the pathophysiological processes of the disease but has failed as a target in early clinical trials [69,70]. Tetrameric NMDARs are regulated by calcium-dependent inactivation (CDI), an event regulated by CaM, but the cellular impact of CDI and the kinetics of this regulation are complex and still under analysis [71]. Here, we provide a basic overview. Binding of CaM occurs on the C0 and C1 domains of the NR1 subunit increasing receptor inactivation and release from the membrane in response to the influx of calcium [72]. CDI involves both apoCaM and Ca^2+^/CaM binding to appropriate domains close to ion channel pore openings, a juxtaposition that allows a rapid response to changes in calcium ion levels [73,74]. At normal calcium levels, apoCaM binds an IQ-like motif in the C-1 region facilitating ion channel opening [75]. Increases in cytosolic calcium levels convert apoCaM to Ca^2+^/CaM causing it to translocate to the Ca^2+^-dependent CaMBD causing CDI. There are multiple NMDAR variants, each which is affected differentially by these events. Basically, a significant increase in cytosolic calcium levels activates Ca^2+^/CaM binding to the channel shutting down any further calcium influx [71]. NMDARs have been well studied and are a meaningful TBI therapeutic target that is still under study.

VGCCs were originally characterized by their biophysical and pharmacologic properties as L-, N-, P/Q- or N-types. A revised gene-based nomenclature is now used, so L-type channels are Cav1.1–1.4, P/Q is Cav 2.1, N is Cav 2.2, R is Cav 2.3 and T-type are Cav3.1–3.3 [76,77]. So, as done here, early articles use the old system while more recent papers tend towards the new classification system. As seen for NMDAR, the influx of calcium through VGCCs is also regulated by calmodulin-regulated CDI [73]. The binding of CaM to all VGCCs is highly conserved and occurs via both calcium-independent and calcium-dependent binding domains [78,79]. A calcium-independent C-term IQ motif (I/L/V)QXXXRXXXX(R/K) and a calcium-dependent N-term CaMBD (xWxxx(I or L)xxxx) have been experimentally validated [78,79]. At low neuronal calcium levels, apoCaM binding keeps the VGCC calcium channels open allowing influx of the divalent cation, but with increased levels of calcium, Ca^2+^/CaM binding inhibits calcium movement through VGCCs. Thus, VGCCs have been and continue to be useful targets for initial TBI therapy.

To modulate calcium homeostasis, NCX family members transport Ca^2+^ into or out of the cell in response to the electrochemical gradients of Na^+^ and Ca^2+^, events that are regulated by CaM [80]. CaM senses cytosolic changes in calcium and responds by binding via a 1-5-8-14 CaMBD to the cytoplasmic loop of NCX1. Similar to other ion channel regulation, CaM binding enhances ion transport by inserting channels into the plasma membrane and removing the autoinhibitory domain in NCX1 and other NCX family members [80,81]. Although evidence indicates TBI results in a decrease in calcium extrusion by NCX, their complex multiple ion exchange role suggests they would be a poor therapeutic target.

## 7. CaMKII, Calcineurin and TBI

The protein kinase CaM-dependent protein kinase II (CaMKII) and protein phosphatase 2B calcineurin (CN, PP2B), each of which is regulated by CaM, often serve contrasting collaborative roles as exemplified by their functions in memory formation [82]. As the primary CBP in neurons, CaM binds to and regulates CaMKII, the major neuronal kinase. Existing as many isoforms, CaMKII functions in the symptomology of TBI. Increased calcium activates CaM which in turn binds to and increases the activity of CaMKII [37,83,84]. Typically activated after TBI, CaMKII has been shown to cause the desensitization of NMDARs and increase the number of AMPA receptors in the cell membrane thus contributing to increased intraneuronal calcium levels associated with the disease [37,83]. CaMKII also binds to and phosphorylates Cav1.2 (L-type VGCC) which is central to TBI [84]. CaMKII has been shown to have these same functions in neurodegenerative diseases [85]. Increased calcineurin activity, as found in the rat hippocampus after TBI, can also contribute to the long-term effects of TBI via its ability to prolong astrocyte activation, an event involved in neuroinflammation [86,87]. Calcineurin dephosphorylates AMPA receptors leading to their increased accumulation in neuronal cell membranes where they work with NMDAR to regulate cytosolic calcium [88]. That said, calcineurin also has multiple downstream functions that need further research to understand its role in the later events of TBI but research on neurodegeneration provides additional insight. For example, hyperactivation of calcineurin in the hippocampus has been observed in models of AD and has been linked to impaired memory, increased tau phosphorylation and neuronal death [89,90]. In plaque-bearing YFP-APP/PS1 transgenic mice, a model for AD, inhibition of calcineurin with the immunosuppressive drug Tacrolimus (FK506) effectively reversed object recognition deficits while reducing plaque burden and improving dendritic spine loss and neuronal integrity [91]. Would FK506 treatment be a viable early choice for treating initial TBI injury? Other CaMBPs are also linked to later events of TBI but are beyond the focus of this review. For example, the biomarker neurofilament light chain, levels of which appear early, serves as an indicator of axonal pathology [92]. In summary, CaMBPs, especially CaMKII and CN, could be useful downstream therapeutic targets for the treatment of TBI.

## 8. Discussion

Since a single concussive event can progress to long-term symptoms and cognitive decline, beginning therapeutic treatment immediately could potentially prevent most cases of PCS [19]. At present, there are no effective treatment protocols for TBI [20,21]. Here, data have been presented that the disruption of calcium levels in brain cells is one of the earliest, if not an initial, cellular event in mTBI that leads to changes that can further increase damaging amounts of calcium that underlie the long-term symptomology characterizing different types of TBI. As detailed above, despite the fact one-third of mTBI cases progress to PCS which can lead to neurodegenerative diseases, the current medical approach for mTBI is rest and non-prescription pain medication [15,16]. In short, currently there is no widely accepted protocol for dealing with the initial effects of mTBI.

## 9. mTBI: A Therapeutic Proposal

**Figure 4:**
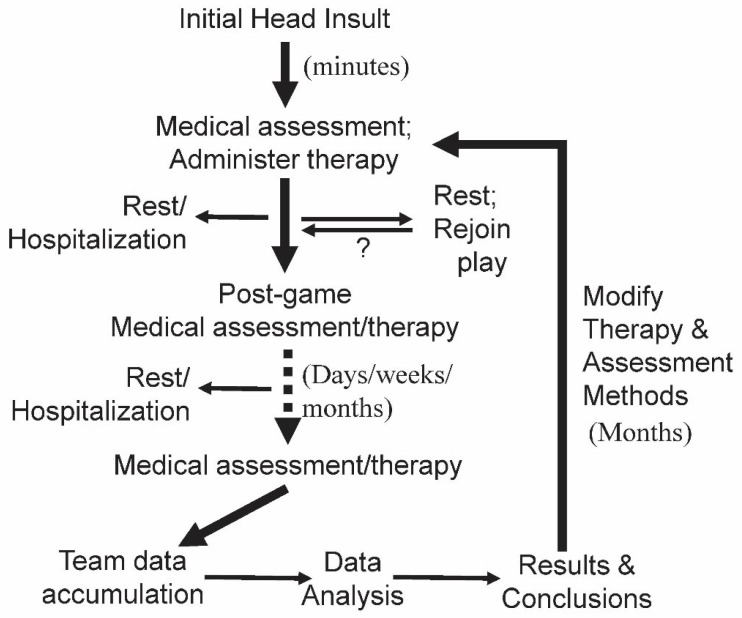
Working outline for a proposed strategy to treat the initial insult of mild TBI. Based on a medical assessment carried out by the team doctor or other professional medical personnel, the chosen therapy is immediately administered after an initial head insult to a member of a professional sports team. As would normally occur, an assessment about the ability to return to play or to undergo rest or hospitalization would be determined. Notes would be made for future reference and data analysis. After the game, a post-game assessment would determine if further treatment is required. The player would continue to be monitored over the next days and weeks with appropriate medical decisions made and therapies applied. After a month or so, a final medical assessment would be made for the individual. At a specified interval, the team data for all mTBI cases would be accumulated and sent to a central area for comprehensive analysis of data from all teams involved in the program. The results would define the next cycle of therapeutic treatment.

While tests (e.g., evaluating biomarker levels, cognitive testing) need to be employed with progressive symptoms and can give some indication of the significance and potential outcome of a TBI event, they take time to implement. Rather than wait for such data and allowing any initial damage to become worse, it would be best to initiate treatment immediately. Here, a working proposal is made for using organized sports to assess therapies to immediately treat the effects of mTBI with the goal of protecting players from future serious symptoms and provide data that could also be of value to the general public (Figure 4). Since there is an extensive literature implicating the early disruption of calcium signaling by mTBI, some potential treatments based on this are covered below.

In the search for understanding the earliest events of TBI and the potential for developing therapies a number of issues and informational deficiencies remain. There are strong arguments that research into finding a cure for TBI-induced symptoms has not been as rigorous or focused as it could be [17,20]. During the last two decades, more than two dozen professional TBI guidelines have been developed or updated but so far they have been unsuccessful [21]. One potential problem is that delaying formal treatment for hours, days or weeks allows basic, potentially controllable changes to become neurologically overwhelming. This is the primary argument for initiating therapies immediately after the initial mTBI insult. The vast majority of organized sports-related (e.g., football, ice hockey, soccer, baseball) TBI is non-invasive mTBI. Professional team sports present unique groups for the development of TBI therapies since they offer multiple levels of control and precision, not available to other mTBI subpopulations. For professional sports this includes immediate medical help, medical and physical records for players and, usually, immediate access to medical treatment facilities. Evidence has shown that mTBI can progress to PCS with a certain frequency that can be evaluated after therapeutic intervention or the lack of treatment [18]. Since most professional athletes will be relatively healthy individuals on substantial diets, many secondary issues that can affect results will be minor compared to the general public. Thus, it can be argued that organized sports, especially professional groups, could serve as excellent cohorts to test and evaluate early therapeutic treatments for mTBI. What is more, data could be evaluated to reveal the short-term (days, weeks) and long-term (months, years) effects of both untreated and treated TBI.

After the initial treatment, players would be assessed using a select group of tests (e.g., Glasgow Outcome Scale—Extended (GOSE), 12-Item Short-Form Health Survey, version 2 (SF-12v2) and/or others) [18]. Team medical personnel would administer the therapy, keep records, administer tests and provide the data to an outside evaluation group such as the International Mission for Prognosis and Analysis of Clinical Trials in TBI (TBI-IMPACT.org). While this adds another level of complexity to team organizations, interest in participating in program should be high since it the program would provide invaluable data to ensure the continued health of existing players, information to protect their players in future and help with the problem of TBI worldwide, not to mention the public support they would receive for such a forward move. The more extensive and precise data from such studies would also be useful for return to play assessment, ensuring that players are fit and not in danger of aggravating any existing TBI-related conditions that could impact the team or the player’s career [93]. The extensive and cumulative data from these studies would also be helpful in assessing gender-based changes in the incidence of TBI in different sports an issue that so far has been challenging “due to the lack of consistency in reporting” [94].

## 10. Some Potential Treatments

Selecting the most appropriate and safe drug that can be given immediately after an initial head impact but which would not otherwise affect the players safety and ability to continue play, if deemed reasonable by a team doctor, will require detailed evaluation. While a specific drug to stop calcium dysregulation does not exist, there has been success targeting specific calcium ion channels and receptors with pharmaceuticals that fulfill these requirements. The current state of the art would argue that human treatments with calcium ion antagonists, especially VGCCs, could begin immediately. There is no shortage of calcium ion channel blockers; the key is to decide which ones would be most appropriate. Since there is an early and intense increase in calcium levels immediately after the initial head insult and since these levels continue to increase then it seems a robust and general shutdown of channels that lead to this increase is in order. In short, stop the disruptive levels of calcium in their tracks, in turn, opening the door for longer-term symptom-specific therapies if they become required. As discussed above, L-type VGCCs have been linked to TBI and the L-type CaV1.2 isoform appears to offer a primary target for earliest therapeutic intervention. CaV1.2 channels are highly expressed in the brain where they localize to the soma and dendrites of most neuron types [95,96]. CaV1.2 is a risk factor for bipolar disorders, schizophrenia, post-traumatic stress syndrome and other neurotraumatic disorders. The dihydropyridine isradipine which inhibits both CaV1.2 and 1.3 has been used in the treatment of schizophrenia and Parkinson’s disease, for which it is now undergoing clinical trials [95]. On the other hand, many L-type VGCC inhibitors currently employed in clinical practice, including isradipine, verapamil and diltiazem, each of which shows a higher affinity for CaV1.2 than CaV1.3 channels, should also be considered [95]. Although VGCCs may be the best target, therapeutic approaches have also been employed targeting NMDARs [43]. Memantine, an NMDAR antagonist that reduces the rate of calcium influx, provides some symptomatic relief in patients with moderate to severe AD [97].

While targeting calcium dysregulation is the prudent and more acceptable approach, the longer-term goal of finding more precise calcium-regulated targets would also benefit from this therapeutic approach of treating professional athletes to prevent the early and progressive symptoms of TBI. As detailed above, CaM is a primary target for calcium increases and multiple CaMBPs have been linked to TBI including VGCCs, NMDAR and enzymes that target them including CaMKII and CN. A diversity of medically approved CaM antagonists exist, as do specific inhibitors for CaMKII and CN. Therapies using safe and effective CaM antagonists have been employed for decades. For example, trifluoperazine and tamoxifen have been used to treat pancreatic and other cancers [98,99]. Polysialic acid-based micelles, that effectively cross the blood–brain barrier, have been used to deliver the CaM antagonist DY-9836 to treat vascular dementia [100]. Quercetin, a flavonoid CaMBP, is undergoing testing in a Phase 2 AD clinical trial [101]. Specific CaMBPs have also been targeted. Tacrolimus (FK506), an immunosuppressant that inhibits calcineurin, is used after transplant surgery to prevent organ rejection [102]. It has also been used to reduce plaque levels, restore memory and reduce Alzheimer’s dementia in humans and mice [102,103]. Pharmaceuticals that target different states of CaMKII, an important CaMBP in neuroinflammation and neurodegeneration, are available and their human uses are being evaluated [104]. Curcumin, which binds CaMKII, is being evaluated in one Phase 2 AD clinical trial [101]. A recent review suggests that curcumin treatments offer protective effects against TBI in different animal models but developing new derivatives could enhance its therapeutic value [105]. The multitude of therapeutic anti-inflammatory and antioxidant benefits of curcumin and the cellular pathways regulated by it have been reviewed, but more human research is required [106,107]. Other approaches to shutting down CaMKII function include antisense oligonucleotides, small interfering RNAs and miRNAs [104]. It might be more realistic to target multiple components. Thus, targeting CaM and/or one or more specific binding proteins could be a valid therapeutic approach to dealing with initial head injuries to prevent the subsequent potentially devastating events of TBI.

The general proposal outlined here will require fine tuning and proper implementation, but it sets the stage for a scheme for immediately dealing with the devastating impact of mTBI in organized sports and for using the results to treat all cases of mTBI. Furthermore, this proposal does not preclude the continued development and evaluation of novel drugs through the use of model systems, a critical process in drug development, or studies on understanding and relieving the long-term impact of PCS.

## Data Availability

Not applicable.
